# Zoonotic approach to Shiga toxin-producing *Escherichia coli*: integrated analysis of virulence and antimicrobial resistance in ruminants and humans

**DOI:** 10.1017/S0950268819000566

**Published:** 2019-03-26

**Authors:** B. Oporto, M. Ocejo, M. Alkorta, J. M. Marimón, M. Montes, A. Hurtado

**Affiliations:** 1 NEIKER – Instituto Vasco de Investigación y Desarrollo Agrario, Animal Health Department, Bizkaia Science and Technology Park 812L, 48160 Derio, Bizkaia, Spain; 2 Hospital Universitario de Donostia, Paseo Doctor Beguiristain, 109, 20014 Donostia, Gipuzkoa, Spain

**Keywords:** Antimicrobial resistance (AMR), cattle, haemolytic uremic syndrome (HUS), sheep, Shiga toxin-producing *Escherichia coli* (STEC)

## Abstract

In 2014–2016, we conducted a cross-sectional survey in 115 sheep, 104 beef and 82 dairy cattle herds to estimate Shiga toxin-producing *Escherichia coli* (STEC) prevalence, and collected data on human clinical cases of infection. Isolates were characterised (*stx*1, *stx*2, *eae*, *ehx*A) and serogroups O157 and O111 identified by PCR, and their antimicrobial resistance (AMR) profiles were determined by broth microdilution. STEC were more frequently isolated from beef cattle herds (63.5%) and sheep flocks (56.5%) than from dairy cattle herds (30.5%) (*P* < 0.001). A similar but non-significant trend was observed for O157:H7 STEC. In humans, mean annual incidence rate was 1.7 cases/100 000 inhabitants for O157 STEC and 4.7 for non-O157 STEC, but cases concentrated among younger patients. Distribution of virulence genes in STEC strains from ruminants differed from those from human clinical cases. Thus, *stx*2 was significantly associated with animal STEC isolates (O157 and non-O157), *ehx*A to ruminant O157 STEC (*P* = 0.004) and *eae* to human non-O157 STEC isolates (*P* < 0.001). Resistance was detected in 21.9% of human and 5.2% of animal O157 STEC isolates, whereas all non-O157 isolates were fully susceptible. In conclusion, STEC were widespread in ruminants, but only some carried virulence genes associated with severe disease in humans; AMR in ruminants was low but profiles were similar to those found in human isolates.

## INTRODUCTION

Shiga toxin-producing *Escherichia coli* (STEC) are important human pathogens that can cause a variety of clinical manifestations, from self-limited diarrhoea to more severe illness such as haemorrhagic colitis or haemolytic uremic syndrome (HUS) [1]. Hence, STEC is the most common infectious agent causing HUS, a syndrome characterised by progressive renal failure. HUS is associated to high morbidity and mortality during the acute phase and to long-term renal and extrarenal complications, particularly in children [2]. Ruminants, and cattle in particular, are considered the primary reservoir of STEC, and the main source of human infection [3]. Contamination of food of animal origin can occur during milking, slaughtering and evisceration. Moreover, faecal shedding of STEC by infected animals can contaminate the environment of the farm, where STEC may survive for long periods of time [4]. Contaminated manure used as fertiliser or effluents from farmland containing STEC can also contaminate fresh produce [5].

STEC are distinguished from non-pathogenic *E. coli* strains isolated from the normal intestinal microbiota of healthy mammals by the production of one or more variants of Shiga toxin (Stx), encoded by *stx*1 and/or *stx*2 genes. The presence of *stx*2 has more frequently been associated with severe disease [6]. Aside from the production of Stx, STEC have other potential virulence factors such as the intimin, an outer membrane protein encoded by the *eae* gene that facilitates intimate attachment to intestinal epithelial cells thereby increasing disease severity [7], and the plasmid-encoded enterohaemorrhagic *E. coli* haemolysin (EhxA)[8]. Several O serogroups have been found associated with Stx production, but O157:H7 is considered the most pathogenic serotype and is responsible for most HUS cases of bacterial aetiology worldwide [9]. In addition, other non-O157 STEC serogroups have also been implicated in severe disease forms as, for example, O111, a serogroup associated with HUS and frequently incriminated in outbreaks in Spain [10] and worldwide [11].

In Spain, declaration of human STEC isolations has only recently become compulsory (Order SSI/445/2015), as per European Union legislation, making the reporting of human STECs to European Food Safety Agency (EFSA) only partial and based on sentinel surveillance so that no information on estimated coverage and notification rate can be estimated. Furthermore, the monitoring programmes of STEC in ruminants are performed only in young beef cattle (1–2 years old) at slaughterhouses selected from different regions of Spain to represent the total volume of cattle sacrificed in the country. As such, small regions like the Basque Country (northern Spain) are not well represented and so the real prevalence in livestock of STEC in the Basque Country cannot be inferred from those surveys. Furthermore, the last study performed regarding livestock incidence of STEC in the Basque Country was carried out in 2003–2005 and in that study STEC was shown to be widespread in cattle and sheep [12]. Moreover, notification compliance rate of human STEC cases in the Basque Country has been traditionally higher than in other regions and comparable data are available in annual reports. Based on those reports, the incidence of STEC infection in humans has been increasing over the years (0.28 in 2004 [13], 0.51 in 2015 [14]) culminating in an incidence of 1.11 cases/100 000 inhabitants in 2016 [15]. The absence of recent studies regarding the prevalence of STEC in livestock precludes the inference of the contribution of livestock in the increase of STEC human cases in our region.

The use of antibiotics in the treatment of STEC infections is controversial and not recommended according to the current clinical guidelines, as certain antimicrobials can induce Shiga toxin production [16, 17]. However, antimicrobial resistance (AMR) is a subject of growing concern due to the widespread of *E. coli* resistant to all antibiotics used in human therapy, and the dissemination of resistance through mobile genetic elements. A recent example is the detection in China of plasmid-mediated antibiotic resistance to colistin [18], one of the last resource antibiotics for the treatment of multi-drug-resistant *Enterobacteriaceae* in humans, which has recently been also isolated in cattle in Spain [19]. Thus, systematic monitoring of the occurrence of AMR in both commensal and pathogenic *E. coli*, like STEC, is essential to measure the spread of resistance among food-borne bacteria and design and implement new control strategies [20].

In this context, the objectives of the present study were (i) to update herd-level prevalence estimates of STEC in their main animal reservoirs (cattle and sheep) in the Basque Country; and (ii) to assess the potential health risk for humans of livestock-carrying STEC through the analysis and comparison of virulence and AMR profiles of human and ruminants STEC strains.

## METHODS

### Sampling design for the estimation of STEC prevalence in ruminant farms

A cross-sectional survey was carried out to estimate the prevalence of STEC in cattle herds and sheep flocks in the Basque Country (Northern Spain). The sampling strategy has been previously described [21], as this study is part of a larger one that intends to determine the prevalence of *Salmonella*, *Listeria monocytogenes* [21] and thermophilic campylobacters (unpublished data) in ruminant farms. Briefly, the number of herds of beef cattle, dairy cattle and sheep farms to be sampled was determined based on the official census for an expected herd prevalence of 50%, a 95% confidence level and an accuracy of 10% using Win Episcope 2.0. Thus, a total of 301 herds (115 dairy sheep, 104 beef and 82 dairy cattle) were sampled once between February 2014 and June 2016. Data on herd size were collected. Rectal faecal samples from 25 animals randomly selected per herd were collected with a gloved hand, and a 25 g pool was prepared (1 g per animal per pool) for microbiological analyses. Sample collection was carried out by veterinary practitioners as part of the usual screening scheme performed on farms, strictly following Spanish ethical guidelines and animal welfare regulations (Real Decreto 53/2013). The collection of this material, being considered routine veterinary practice, did not require the approval of the Ethics Committee for Animal Experimentation. Informed oral consent was obtained from the farm owners at the time of sample collection. Details on general husbandry systems for beef cattle, dairy cattle and sheep in the Basque Country were reported elsewhere [21].

### STEC isolation from animal faeces and strain characterisation

Faeces (25 g of pooled faecal samples) were diluted 1:10 in modified Tryptic Soy Broth (mTSB, BioMérieux) supplemented with novobiocine (Biolife) and incubated at 41 ± 1 °C for 6–7 h. The mTSB broth was subcultured onto MacConkey agar at 37 ± 1 °C for 24 h. To detect STEC, 10 lactose-positive colonies from the MacConkey agar were analysed in two pools of five in a multiplex Real-Time PCR reaction targeting the genes encoding the Shiga toxin 1 and 2 (*stx*1 and *stx*2) and the *E. coli* attaching and effacing gene (*eae*). For *stx*-positive pools, colonies were individually tested to confirm their identity as STEC using the same multiplex Real-Time PCR. Primers and probe for *eae* were as previously described [22], whereas those for *stx*1 and *stx*2 were based on those described by Perelle *et al*. [23] with slight modifications in the sequence of the primers (*stx*1-F: 5′-TTTGTTACTGTGACAGCTGAAGCTTTA-3′; *stx*1-R: 5′-CCAGTTCAATGTAAGATCAACATCTTC-3′; *stx*2-F: 3′-GTCACTGTCACAGCAGAAGCCTTA-5′; *stx*2-R: 5′-CAGTTCAGAGTGAGGTCCACGTC-3′). One STEC isolate from each positive herd/flock was selected for further analyses, with the exception of herds/flocks where more than one *stx*1/*stx*2/*eae* profile was found. Further characterisation included the amplification of the genes encoding the O157 somatic antigen (*rfb*E), the H7 flagellar antigen (*fli*C) and the enterohaemolysin (*ehx*A) in a conventional multiplex PCR [24], and the O111 serogroup-related gene *wzx*O111 [25].

The detection of O157 STEC was performed as follows: DNA was extracted directly from the bacterial culture obtained from MacConkey agar plates and screened for the presence of the *rfb*E gene by Real-Time PCR as previously described [23]. PCR-positive samples were then submitted to immunomagnetic separation (IMS) using magnetic Dynabeads anti-*E. coli* O157 (Dynal, Oslo, Norway), followed by chromogenic isolation (chromID™ O157:H7/NM agar with CT, BioMérieux). Typical bluish-green colonies (two per sample) were considered presumptive *E. coli* O157 and confirmed by Real-Time PCR amplification of *rfb*E as described above. Confirmed *E. coli* O157 colonies were characterised for the presence of *stx*1/*stx*2/*eae* (multiplex Real Time PCR) and *fli*C and *ehx*A (conventional PCR) using the abovementioned procedures for STEC.

Minimum inhibitory concentrations (MIC) were determined by broth microdilution using a Sensititre^®^ MIC Susceptibility Plates (EUVSEC1, ThermoFisher Scientific, Waltham, MA, USA) containing 14 antimicrobial agents (12 classes) following recommendations by the Commission Decision 2013/652/EU. Results were interpreted using epidemiological cut-off values based on the distribution of MICs of wild-type susceptible populations as developed by the European Committee for Antimicrobial Susceptibility Testing (EUCAST, http://www.eucast.org).

### STEC isolation from human stool samples and strain characterisation

A total of 29 094 stool samples from inpatients and outpatients of all ages sent to the Microbiology Service of *Hospital Universitario Donostia* between January 2014 and December 2016 with symptoms of gastrointestinal disease were screened for the presence of STEC. Following routine procedures, DNA was automatically extracted from stool samples (NUCLISENS^®^ EASYMAG^®^, BioMérieux) and analysed using a real-time multiplex PCR kit (FTD Bacterial gastroenteritis, Fast Track diagnostic^®^, Werfen) that detects *Salmonella* spp., *Shigella* spp., *Yersinia enterocolitica*, *Clostridium difficile*, *Campylobacter* (*C. coli*/*C. jejuni*/*C. lari*) and STEC (*stx*+). Stx-positive stool samples were cultured onto SMAC and MacConkey agar (BioMérieux) and incubated at 37 ± 2 °C for 24–48 h. Typical sorbitol-negative *E. coli* O157 colonies on SMAC agar were identified by MALDI-TOF and confirmed by O157-specific slide agglutination using the *E. coli O157 Latex Test Kit (Oxoid)*, and further characterised by PCR for the presence of *stx*1, *stx*2, *eae*, *ehx*A and *rfb* O157 as previously described [26]. One lactose-positive colony per MacConkey agar plate was also analysed by MALDI-TOF, and those colonies identified as *E. coli* were further characterised for the presence of *stx*1, *stx*2, *eae*, *ehx*A, *rfb* O111 and *rfb* O157 by PCR as described above. Patient ages were recorded for all STEC-positive cases. For patients with STEC O157 or O111 infection, data on hospitalisation, clinical evolution, focused on the development of HUS and outcome were also recorded. The AMR profile of O157 and O111 isolates was determined using the Vitek 2 system (BioMérieux) and the AST-N244 (Gram-negatives) card according to the manufacturer's instructions and clinical cut-off values were applied according to CLSI guidelines [27].

### Statistical analysis

Herd-level prevalence estimates were expressed as the percentage of herds/flocks that tested positive in each farm system out of all herds/flocks that were examined in the respective farm system, with 95% confidence intervals adjusted for the population size, using the software EpiInfo2. Differences in prevalence frequencies were assessed using statistical software Stata/IC version 13.1 (StataCorp LP, College Station, TX, USA). Association between variables of interest and proportion of ruminant herds shedding STEC and *E. coli* O157:H7 was assessed. Variables tested were: (i) farm management system (sheep, beef cattle and dairy cattle), (ii) animal species (sheep and cattle), (iii) sampling season (categorised according to calendar year, i.e. spring, summer, autumn, winter), (iv) geographical location of the farm (oceanic, continental) and (v) herd size stratified according to farm system management as follows: beef cattle,<50, 50–100 and>100; dairy cattle,<50, 50–150 and>150; sheep,<150, 150–300 and>300. Analyses were first performed in the whole dataset to test the association of each of the variables with STEC and *E. coli* O157:H7 farm prevalence. Then, data were stratified to the variables associated with statistically significant differences. Annual incidence rate of human clinical cases was calculated as the number of cases accumulated in the 3 years for each age group divided by the population size at risk (i.e. population size for each age group multiplied by 3 years at risk of event), and then referred to 100 000 inhabitants. The effect of seasonality in *E. coli* O157:H7 and non-O157 human infections was evaluated with data stratified by age (infants, 0–5 years; teenagers, >5–15 years; adults, >15–65 years; elderly, >65 years). The distribution of the different virulence genes and profiles of O157 and non-O157 STEC isolates from different sources was compared. Thus, virulence genes distribution (presence or absence) was compared between isolates from animal–human, cattle–human, sheep–human, cattle–sheep and dairy cattle–beef cattle. For virulence genes profiles, variables were stratified to two categories (the category of interest and all other categories) and its presence compared between isolates of animal and human origin. One sample test of proportions with 95% CI was performed to look for differences in *eae* gene carriage among non-O157 human clinical isolates. The *χ*^2^ or Fisher's exact tests were run, depending on sample size and expected frequencies [28]. In all cases (animal and human), when overall significance was < 0.05, post-hoc analysis involving pairwise comparisons of two proportions was performed. Bonferroni correction was applied to compensate for multiple groups testing (to control Type I error) and statistically significant differences were determined based on adjusted *P*-values (*P*-value 0.05 divided by the total number of pairwise comparisons).

## RESULTS

### Prevalence of STEC-positive herds/flocks

The distribution of herds positive to STEC (any serotype) and *E. coli* O157:H7 by host animal species is shown in Table 1. STEC were more frequently isolated from beef cattle (63.5% of herds) and sheep (56.5% of flocks) than from dairy cattle (30.5% of herds) (*χ*^2^ = 13.07, *P* < 0.001 and *χ*^2^ = 19.95, *P* < 0.001, respectively). STEC serogroup O157 were only isolated when IMS was performed, and all isolates identified as *E. coli* O157 carried the H7 flagellar antigen gene *fli*C, thus confirming the strains to be serotype O157:H7. STEC serogroup O157 were more frequently isolated from sheep flocks (20.0%) and beef cattle herds (18.3%) than from dairy cattle herds (14.6%) but differences were non-significant (*P* = 0.623). Only in the case of sheep flocks, differences in *E. coli* O157:H7 prevalence were found between sampling seasons (*χ*^2^ = 8.08, *P* = 0.044); prevalence in summer and autumn being higher than winter (*P* = 0.027 and *P* = 0.016, respectively). However, these differences were no longer significant after Bonferroni correction. STEC serogroup O111 was not detected in this study. Regarding herd size, no significant associations with STEC (any serotype), O157 STEC or non-O157 prevalence (*P* > 0.05) were observed when considering all farms together. When comparisons were stratified by farm system, only beef cattle showed differences in O157 STEC excretion, with medium-sized herds presenting higher rates of O157 STEC than small farms (*χ*^2^ = 7.361, *P* = 0.007). No significant associations were found between STEC shedding and any of the other variables tested.

**Table 1.** Herds/flocks positive to Shiga toxin-producing *Escherichia coli* (STEC) (any serotype), O157:H7 STEC and non-O157 STEC

Animal source	Analysed herds	STEC		O157:H7 STEC		Non-O157 STEC	
		*n*	% (CI)	*n*	% (CI)	*n*	% (CI)
Beef cattle	104	66	63.5 (54.5–72.5)	19	18.3 (11.0–25.6)	61	58.7 (49.5–67.9)
Dairy cattle	82	25	30.5 (21.5–39.5)	12	14.6 (7.7–21.5)	16	19.5 (11.8–27.2)
Sheep	115	65	56.5 (48.4–64.6)	23	20.0 (13.5–26.5)	53	46.1 (38.0–54.2)

### Characterisation of virulence genes of STEC isolated from ruminants

**Fig. 1.**
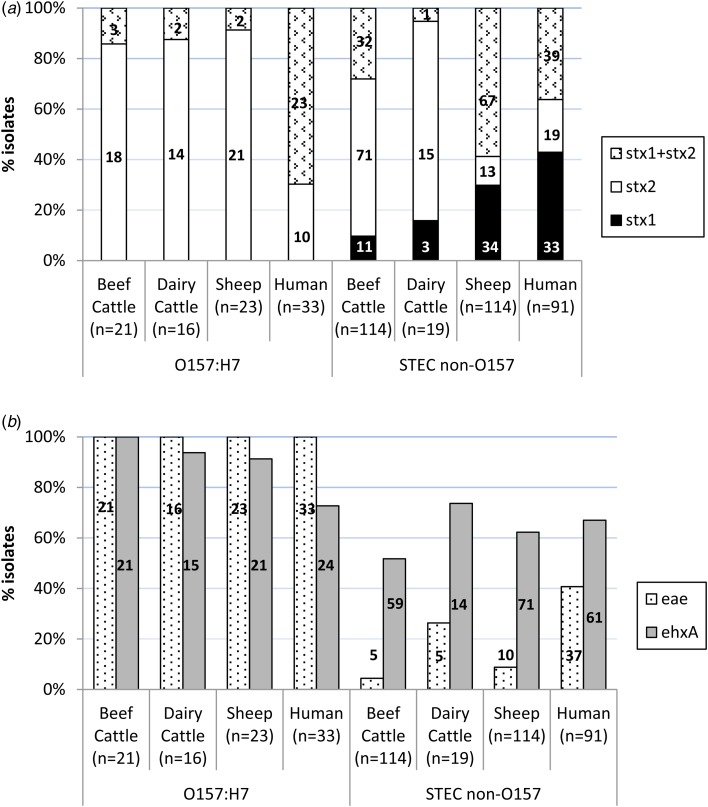
Distribution of virulence genes among Shiga toxin-producing *Escherichia coli* from ruminants (307 isolates from 156 herds) and human clinical cases (124 isolates). (A) Shiga-toxin genes; (B) *eae* and *ehx*A genes. Number of isolates is indicated within the columns.

An average of two colonies (maximum six) per STEC-positive herd/flock were characterised for the presence of *stx*1, *stx*2, *eae* and *ehx*A, amounting to a total of 307 STEC isolates from different sources (135 isolates from 66 beef cattle herds, 35 isolates from 25 dairy cattle herds and 137 isolates from 65 sheep flocks) and serogroups (60 O157:H7 and 247 non-O157). Eleven different patterns of virulence gene combinations were found in STEC from ruminants (Table 2). However, only three profiles were observed among the O157:H7 isolates, with the toxigenic profile *stx*2 + *eae* + *ehx*A as the most prevalent (50/60, 83.3%), but very uncommon (4/247, 1.6%) among non-O157 STEC (Table 2). All *E. coli* O157:H7 strains carried the *stx*2 and *eae* genes, 95.0% (55/60) also carried the *ehx*A gene and 11.7% (7/60) carried the *stx*1 gene (always in combination with *stx*2) (Fig. 1). No differences were found regarding the prevalence of genes in *E. coli* O157:H7 isolated in the different animal species analysed (*P* > 0.05). Conversely, the distribution of Shiga toxin genes was clearly different in non-O157 isolates collected from sheep and cattle (Table 2, Fig. 1A). Hence, *stx*2 gene alone was rarely found in sheep isolates (13/114, 11.4%), which more frequently carried *stx*1 and *stx*2 genes (67/114, 58.8%); whereas in cattle, *stx*2 gene alone was more prevalent (86/133, 64.7%). All comparisons of Shiga toxin gene distribution in non-O157 STEC between cattle and sheep were statistically different (*P* < 0.001). Presence of *eae* and *ehx*A genes among non-O157 isolates was not as widespread as in O157:H7 isolates (Fig. 1B), and no differences were found between sheep and cattle (*P* > 0.05) but *eae* was more prevalent in isolates from dairy than beef cattle (*P* = 0.006). The combination of *eae* and *ehx*A genes in non-O157 isolates from ruminants was very rare (13/247, 5.3%). Herds shedding non-O157 STEC-positive for any *stx* gene (*stx*1 and/or *stx*2) and *eae* gene would include four beef cattle herds (3.8%; five isolates), five dairy cattle herds (6.1%; five isolates) and eight sheep flocks (7.0%; 10 isolates). If only the more pathogenic combination *stx*2 + *eae* was considered, the percentage of herds shedding this particular non-O157 STEC genotype would be even smaller (3.8% of beef cattle herds, 3.6% of dairy cattle and 5.2% of sheep flocks).

**Table 2.** Combination of virulence genes among Shiga toxin-producing *Escherichia coli* from ruminants (307 isolates from 156 herds) and human clinical cases (124 isolates)

		O157:H7				Non-O157			
Gene pattern	Total	Beef cattle	Dairy cattle	Sheep	Human	Beef cattle	Dairy cattle	Sheep	Human
	*n* (%)	(*n* = 21)	(*n* = 16)	(*n* = 23)	(*n* = 33)	(*n* = 114)	(*n* = 19)	(*n* = 114)	(*n* = 91)
*stx*1	37 (8.6)					11	1	19	6
*stx*1 + *ehx*A	22 (5.1)							11	11
*stx*1 + *eae*	6 (1.4)							2	4
*stx*1 + *eae* + *ehx*A	22 (5.1)						2	2	18
*stx*2	57 (13.2)					37	3	9	8
*stx*2 + *ehx*A	48 (11.1)					30	9	2	7
*stx*2 + *eae*	11 (2.6)		1	2	1	2	1	2	2
*stx*2 + *eae* + *ehx*A	65 (15.1)	18	13	19	9	2	2		2
*stx*1 + *stx*2	19 (4.4)					4		7	8
*stx*1 + *stx*2 + *ehx*A	98 (22.7)					27	1	56	14
*stx*1 + *stx*2 + *eae*	10 (2.3)				8				2
*stx*1 + *stx*2 + *eae* + *ehx*A	36 (8.4)	3	2	2	15	1		4	9

### Prevalence of STEC in human patients

**Fig. 2.**
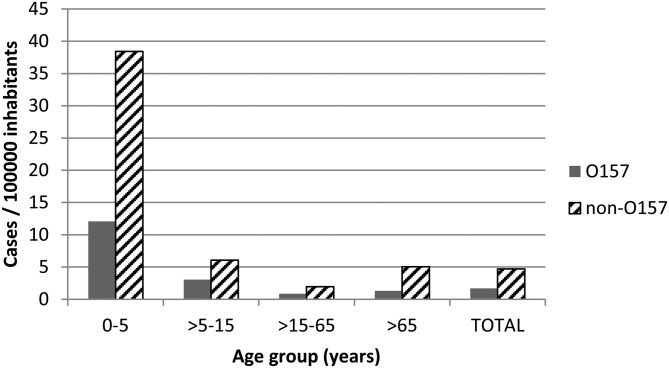
Annual incidence of confirmed STEC (O157 and non-O157) human infections by age.

Of the 29 094 stool samples screened by multiplex-PCR, 233 were *stx*-positive. Culture of the 233 *stx*-positive stool samples yielded 162 isolates confirmed by MALDI-TOFF as *E. coli*, and 124 of them were identified as STEC by individual colony PCR targeting the *stx*1 and *stx*2 genes. Of the 124 STEC, 33 were identified as belonging to serogroup O157, four to serogroup O111 and the remaining 87 isolates belonged to other undetermined non-O157 serogroups. Thus, O157:H7 STEC accounted for 0.11% (33/29 094) cases of gastrointestinal infection and non-O157 STEC for 0.31% (91/29 094). Mean annual incidence rate calculated for the population served by the hospital was 1.7 cases/100 000 inhabitants for O157 STEC and 4.7 cases/100 000 inhabitants for non-O157 STEC, but cases mainly concentrated among young patients (Fig. 2). Actually, of the 33 patients with O157 STEC detected in stool samples, 17 were <12 years old. STEC infections showed a seasonal distribution, with the largest proportion of STEC cases occurring in summer, followed by autumn. In fact, O157 STEC cases concentrated in summer and non-O157 cases in autumn. With stratification by age, these seasonal differences in STEC carriage (both O157 and non-O157) were observed in infants and teenagers (Fisher's exact test, *P* = 0.007 and *P* = 0.023, respectively). However, after pairwise comparisons between seasons, differences were only observed for infants, prevalence of O157 infections in summer being significantly higher than in autumn (*χ*^2^ = 8.044, *P* = 0.005).

The main clinical symptoms included diarrhoea accompanied with vomiting or abdominal pain; in addition, 11 patients had bloody diarrhoea. Twelve patients were admitted to the hospital with a suspicion of gastroenteritis. A 9-year-old boy with stool sample positive for O157 STEC developed HUS that resolved without any sequelae. Another HUS case was observed in a 2-year-old child due to *E. coli* O111 who also recovered without sequelae. Two male patients, aged 57 and 79 years, infected with O157:H7 *E. coli* died; both patients were immunocompromised and had comorbidities.

### Characterisation of virulence genes of STEC isolated from human patients

Distribution of *stx*1, *stx*2, *eae* and *ehx*A in the 124 STEC isolates of human origin is shown in Table 2 and Figure 1. All 12 possible patterns of virulence genes combinations were found in human STEC isolates; these included the 11 virulence gene patterns that had been found in animals plus an additional pattern (*stx*1 + *stx*2 + *eae*) found in both O157 and non-O157 human isolates but not in animals. In human O157:H7 isolates, *stx*1 + *stx*2 + *eae* + *ehx*A was the most common virulence gene profile (15/33, 45.5%) found, followed by *stx*1 + *stx*2 + *ehx*A (27.3%, 9/33). All *E. coli* O157:H7 strains from human clinical cases carried the *stx*2 and *eae* genes, 72.7% (24/33) carried the *ehx*A gene and 69.7% (23/33) the *stx*1 gene. However, human O157:H7 isolates more frequently carried *stx*1 and *stx*2 genes together (23/33, 69.7%) than *stx*2 gene alone (10/33, 30.3%); *stx*1 alone was not observed. Among non-O157 isolates, *stx*1 alone (39/91, 42.9%) was more prevalent than *stx*1 + *stx*2 (33/91, 36.3%) or *stx*2 alone (19/91, 20.9%); *ehx*A was present in 67.0% of the cases (61/91) and *eae* in 40.7% (37/91). Overall, non-O157 human isolates were more likely to be *eae*-negative than *eae*-positive (*P* = 0.037). When stratified by age, this difference was only significant for adults (>15–65 years, *P* = 0.005) and the elderly (>65 years, *P* = 0.006). The combination *stx*2 + *eae* was found in 16.5% (15/91) of non-O157 human clinical isolates. The four serogroup O111 isolates recovered had the following profiles: *stx*1 + *stx*2 + *eae* + *ehx*A (two isolates), *stx*2 + *eae* + *ehx*A (one isolate) and *stx*1 + *eae* (one isolate).

### Comparison of virulence profiles between animal and human STEC

Even though all but one of the virulence profiles described in this study (Table 2) were found in both animals and humans, there were statistically significant differences in the prevalence of virulence genes and profiles in animals and humans. Although in *E. coli* O157:H7 strains, the genes *stx*2 and *eae* were present in all isolates from both ruminants and humans, *stx*2 alone was more prevalent in ruminant than in human isolates (*P* < 0.001) while the combination of *stx*1 + *stx*2 was more frequent in humans (*P* < 0.001). The *ehx*A gene was significantly more prevalent among *E. coli* O157:H7 isolates from animals than humans (*P* = 0.004). Whereas in animal isolates the gene profile *stx*2 + *eae* + *ehx*A was the most prevalent among serotype O157:H7, in human isolates *stx*1 + *stx*2 + *eae* + *ehx*A was the most commonly found. Regarding non-O157 STEC, gene distribution was clearly different between ruminant and human isolates. Hence, the presence of the gene *stx*2 alone was again significantly associated with animal isolates (*P* = 0.001), mainly due to its widespread distribution in cattle, while *stx*1 alone (*P* < 0.001) was significantly associated with human isolates; the combination *stx*1 + *stx*2 did not significantly differ between human and animal isolates considered globally (*P* = 0.526), but it was significantly different when comparing human and sheep isolates (*P* = 0.002). The *eae* gene was significantly associated with human isolates (*P* < 0.001), and the *ehx*A gene was, however, more evenly distributed among the different isolation sources (*P* = 0.115). The highly pathogenic profile *stx*1 + *stx*2 + *eae* + *ehx*A was more frequently associated with non-O157 human clinical isolates (*P* = 0.003), as were two other gene combinations containing *stx*1 gene (*stx*1 + *eae* + *ehx*A, *P* < 0.001; *stx*1 + *ehx*A, *P* = 0.011). Another three virulence gene profiles were more frequently associated with animal isolates (*stx*2, *P* = 0.018; *stx*2 + *ehx*A, *P* = 0.040; *stx*1 + *stx*2 + *ehx*A, *P* = 0.003).

### AMR profiles of STEC isolated from ruminants and humans

AMR profiles were determined for 106 STEC isolates of animal origin (58 O157 and 48 non-O157 isolates representing the 11 virulence genes profiles) and 36 isolates from human clinical cases (32 O157 and four O111). Antimicrobials tested, distribution of MICs and interpretation of results are shown in Table 3. Most of the animal isolates (103/106, 97.2%) were susceptible to all 14 antibiotics tested; only three O157:H7 isolates (two from sheep and one from dairy cattle; gene profiles *stx*2 + *eae* + *hly*A – one isolate, and *stx*1 + *stx*2 + *eae* + *hly*A – two isolates) showed a multiple drug resistance (MDR) profile with resistance to ampicillin, trimethoprim, sulfamethoxazole and tetracycline (Table 4). In the case of human isolates, 80.6% (29/36) were susceptible to all 16 antimicrobials tested, including the two isolates obtained from the patients who developed HUS and the two deceased patients. Seven isolates (all of serogroup O157) were resistant to at least one antimicrobial, and the highest percentage of isolates were resistant to trimethoprim/sulfamethoxazole (16.7%) followed by ampicillin (13.9%). Multidrug resistance phenotypes included resistance to two, three and six antimicrobials (Table 4).

**Table 3.** Resistance (percentage) and distribution of MICs for the 106 ruminant STEC and 36 human STEC (O111/O157) isolates

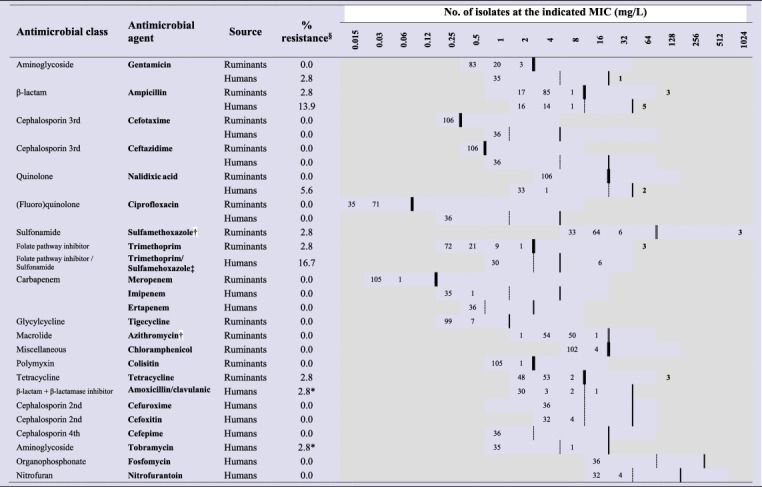

White fields denote range of dilutions tested for each antimicrobial agent. MICs equal to or above the range are given as the concentration closest to the range and indicated in bold. MICs equal to or lower than the lowest concentration tested are given as the lowest tested concentration. Vertical lines indicate cut-off values: EUCAST epidemiological cut-offs (for ruminant isolates) are represented by thicker lines; CLSI clinical cut-offs (for human isolates) in thin lines, dashed for intermediate and continued for resistant.

^a^No cut-off value given by EUCAST; reference as indicated by double vertical lines were used.

^b^Trimethoprim/sulfamethoxazole cut-off values are expressed as trimethoprim concentration (range 1:19–16:304).

^c^All resistant isolates belonged to serotype O157:H7.

^d^Intermediate resistance.

**Table 4.** Resistance phenotypes of STEC isolates

Resistance profile^a^	Source (*n* isolates)
AMP	Human (1)
SXT	Human (2)
AMP/SXT	Human (2)
AMP/SXT/TET	Sheep (2); dairy cattle (1)
AMP/SXT/NAL	Human (1)
AMP/GEN/NAL/SXT/(AMC/TBM^b^)	Human (1)

a AMP, ampicillin; AMC, amoxicillin/clavulanic acid; GEN, gentamicin; NAL, nalidixic acid; SXT, sulfamethoxazole/trimethoprim (tested separately in animal isolates); TBM, tobramycin; TET, tetracycline.

b Intermediate resistance to AMC and TBM.

## DISCUSSION

In this study, we present an updated prevalence estimate of STEC in the main animal reservoirs in the Basque Country along with parallel data on human clinical cases, to contextualise the current risk that ruminants represent to human health and to monitor AMR in the context of a one health approach. The animal survey included not only beef cattle but also dairy cattle and sheep, the latter an important production system in the region for reasons related with traditional and artisan cheese production under Protected Designation of Origin (PDO). Accurate estimates of herd prevalence at each production system were obtained and a collection of strains representative of the region was compiled for characterisation. Data from human clinical cases were obtained from one of the main hospitals of the Basque Country to assure good reporting coverage while collecting a representative number of isolates.

The results of the cross-sectional survey carried out in ruminants showed a high proportion of STEC-positive cattle and sheep herds, at levels similar to those reported in a comparable study carried out in the region in 2003–2005 [12]. Interestingly, and in agreement with the results found in that previous study, the proportion of herds shedding STEC was higher in semi-intensive management systems (beef cattle and sheep) compared with intensive systems (dairy cattle); a similar trend was also observed for O157:H7 STEC. Other authors also found an association between pasture access and STEC shedding [29], but opposite results have also been reported [30, 31]. Animal management or other practices specific to livestock production in different countries might explain these discrepancies [32]. In addition, herd size has also been shown to affect infection prevalence when a higher number of susceptible hosts are continuously challenged by contact with carriers [33, 34]. Here, the effect of herd size was only significant in beef cattle, with medium-sized herds presenting higher rates of O157 STEC than small farms, but reasons are unknown. When considering herds shedding *E. coli* O157:H7, prevalence was higher than that reported in our previous study carried out 10 years earlier, which was estimated to be 8.7% for sheep flocks and 3.8% for cattle herds [12]. However, results from that study were probably an underestimation of the true prevalence due to the methodology used. Still this increasing trend observed in ruminants would be in accordance to the epidemiology of enterohaemorrhagic *E. coli* infections in humans in the Basque Country, which rose from 0.28 to 1.11 cases/100 000 inhabitants in 12 years (2004–2016) [13, 15]. Similarly to the data obtained for animals, the data collected at the *Hospital Universitario Donostia* also showed an increasing trend in STEC incidence, but again, improvements in the methodology used could be one of the main reasons for this apparent increase. In agreement with other studies [35], STEC-associated diarrhoea and HUS cases concentrated among young patients, both for O157:H7 and other non-O157 STEC infections. During the period of this study, two cases of HUS were registered, one caused by *E. coli* O157 in a 9-year-old boy and another caused by *E. coli* O111 in a 2-year-old child, both with a favourable outcome. Serogroup O111 has been one of the main causes of STEC outbreaks in the region [10]; however, it was only isolated in four patients in this study. Also, this serogroup seems to be uncommon in ruminants [10, 36], and, in accordance, no O111-shedding herds were found here. The seasonality of STEC described here, with a larger proportion of cases identified during summer and autumn, agrees with other studies [37–39].

A total of 307 isolates from ruminants were characterised, including 135 from beef cattle, a number that further exceeds those analysed in a nationwide sampling (8 O157 and 56 non-O157) performed in beef cattle in 34 slaughterhouses in Spain in 2011 and 2013 in compliance with Directive 99/2003/EC [40]. An additional strength of this study is that it also included isolates from dairy cattle and sheep, as well as human isolates collected in the same region, allowing comparison of strains from different sources. Interestingly, the virulence gene profiles in *E. coli* O157:H7 isolates, but mainly in non-O157 isolates, were different depending on the isolation host. These could be due to the diversity of non-O157 serotypes isolated from different hosts, but indicates that despite the high proportion of STEC shedding herds, only a small proportion of STEC carried by ruminants could be epidemiologically related to clinical disease in humans. Moreover, despite the wide distribution of genes *stx*2 and *eae* in O157 STEC, a gene combination considered to be more consistently associated with severe disease in humans [41], more than half of patients infected with O157 STEC showed only mild gastrointestinal symptoms. In non-O157 STEC, the *stx*2 gene was more prevalent in ruminant strains (80.6%), particularly in cattle (89.5%), than in human strains (57.1%), but the *eae* gene was present in only 8.1% of the ruminant non-O157 strains, compared with 40.6% of those from human clinical cases. This low prevalence of *eae*-positive isolates among non-O157 from ruminants is in accordance to that found 10 years before [12] and with several other studies [36, 42, 43], but much lower than the 64.3% recently reported in beef cattle in Spain [40]. Although this might suggest that only a small proportion of STEC shedding herds pose a high risk, results from human clinical cases reported here and elsewhere [44] showed that isolates also lacking the *eae* gene can cause gastrointestinal disease. The fact that a significantly higher proportion of non-O157 human isolates were *eae*-negative than *eae*-positive, particularly in adults and the elderly, might indicate that these patients were more frequently infected with serotypes other than the top five non-O157 STEC [45]. Still, all STEC shedding herds should be considered as a potential risk for human infection.

Treatment of human STEC infections is generally supportive and antibiotics are usually contraindicated. However, monitoring AMR patterns of intestinal STEC from healthy ruminants in their intestine and their comparison with those isolated from human clinical cases, provide valuable information of the public health risk posed by animal reservoirs with respect to the transmission of resistant *E. coli* strains to humans and the spread of AMR to other enteric bacteria. Here, the proportion of resistant ruminant isolates was lower than in other studies [40, 43, 46], and only found in O157 STEC. However, all resistant isolates exhibited the same MDR profile (ampicillin/trimethoprim/sulfamethoxazole/tetracycline), consistent with the fact that *β*-lactams, trimethoprim/sulphonamides and tetracyclines are among the most commonly used antibiotics in veterinary medicine [47]. In humans, most isolates tested were O157 strains and 21.9% (7/32) of them showed some level of resistance. Ampicillin resistance was also widespread among human isolates, and all but one of the human O157 isolates with a resistant phenotype were resistant to trimethoprim/sulfamethoxazole. This drug combination is recommended for treating a range of human infections, including infectious diarrhoea when necessary and urinary tract infections (acute uncomplicated cystitis and pyelonephritis) [48], and therefore dissemination of trimethoprim/sulfamethoxazole resistance in *E. coli* isolates should be closely monitored. However, all the isolates were susceptible to fluoroquinolones and third-generation cephalosporins, alternative antimicrobials for treating infections caused by sulfamethoxazole/trimethoprim-resistant *E. coli*. Similarly, all ruminant isolates were susceptible to fluoroquinolones, macrolides, aminoglycosides, third-generation cephalosporins, carbapenems or colistin.

In conclusion, this study represents a thorough survey of STEC infection in ruminants and humans that provided reliable estimates on herd-prevalence data and human incidence rates, and allowed comparison of isolates from different sources. Results showed a wide distribution of STEC in ruminants in the Basque Country, but distribution of virulence factors among STEC strains from sheep and cattle differed from those of human clinical cases. The proportion of resistant isolates in ruminants was not high, but the MDR profiles were similar to those found in human isolates. Therefore, presence of STEC in healthy cattle and sheep represents a potential risk for public health that cannot be neglected.

## References

[1] Karmali MA, Gannon V and Sargeant JM (2010) Verocytotoxin-producing *Escherichia coli* (VTEC). Veterinary Microbiology 140, 360–370. 19410388 10.1016/j.vetmic.2009.04.011

[2] Scheiring J, Andreoli SP and Zimmerhackl LB (2008) Treatment and outcome of Shiga-toxin-associated hemolytic uremic syndrome (HUS). Pediatric Nephrology 23, 1749–1760. 18704506 10.1007/s00467-008-0935-6 PMC6901419

[3] Mughini-Gras L (2018) Attribution of human infections with Shiga toxin-producing *Escherichia coli* (STEC) to livestock sources and identification of source-specific risk factors, The Netherlands (2010–2014). Zoonoses and Public Health 65, e8–e22. 28921940 10.1111/zph.12403

[4] Maule A (2000) Survival of verocytotoxigenic *Escherichia coli* O157 in soil, water and on surfaces. Journal of Applied Microbiology Symposium Supplement 88, 71S–78S. 10.1111/j.1365-2672.2000.tb05334.x 10880181

[5] Solomon EB, Yaron S and Matthews KR (2002) Transmission of *Escherichia coli* O157:H7 from contaminated manure and irrigation water to lettuce plant tissue and its subsequent internalization. Applied and Environmental Microbiology 68, 397–400. 11772650 10.1128/AEM.68.1.397-400.2002 PMC126537

[6] Paton JC and Paton AW (1998) Pathogenesis and diagnosis of Shiga toxin-producing *Escherichia coli* infections. Clinical Microbiology Reviews 11, 450–479. 9665978 10.1128/cmr.11.3.450 PMC88891

[7] Donnenberg MS (1993) The role of the *eae* gene of enterohemorrhagic *Escherichia coli* in intimate attachment in vitro and in a porcine model. Journal of Clinical Investigation 92, 1418–1424. 8376595 10.1172/JCI116718 PMC288286

[8] Lorenz SC (2013) Prevalence of hemolysin genes and comparison of *ehx*A subtype patterns in Shiga toxin-producing *Escherichia coli* (STEC) and non-STEC strains from clinical, food, and animal sources. Applied and Environmental Microbiology 79, 6301–6311. 23934487 10.1128/AEM.02200-13 PMC3811216

[9] Mainil JG and Daube G (2005) Verotoxigenic *Escherichia coli* from animals, humans and foods: who's who? Journal of Applied Microbiology 98, 1332–1344. 15916647 10.1111/j.1365-2672.2005.02653.x

[10] Mora A (2011) Characteristics of the Shiga-toxin-producing enteroaggregative *Escherichia coli* O104:H4 German outbreak strain and of STEC strains isolated in Spain. International Microbiology 14, 121–141. 22101411 10.2436/20.1501.01.142

[11] Piercefield EW (2010) Hemolytic uremic syndrome after an *Escherichia coli* O111 outbreak. Archives of Internal Medicine 170, 1656–1663. 20937925 10.1001/archinternmed.2010.346

[12] Oporto B (2008) *Escherichia coli* O157:H7 and non-O157 Shiga toxin-producing *E. coli* in healthy cattle, sheep and swine herds in northern Spain. Zoonoses and Public Health 55, 73–81. 18234025 10.1111/j.1863-2378.2007.01080.x

[13] Anon (2004) *Informe de Salud Pública del* 2004. Departamento de Sanidad, Gobierno Vasco.

[14] Anon (2015) *Informe 2015: Salud Pública y Adicciones*. Departamento de Salud, Gobierno Vasco.

[15] Anon (2016) *Informe 2016: Salud Pública y Adicciones*. Departamento de Salud, Gobierno Vasco.

[16] Wong CS (2000) The risk of the hemolytic–uremic syndrome after antibiotic treatment of *Escherichia coli* O157:H7 infections. New England Journal of Medicine 342, 1930–1936. 10874060 10.1056/NEJM200006293422601 PMC3659814

[17] Kimmitt PT, Harwood CR and Barer MR (2010) Toxin gene expression by Shiga toxin-producing *Escherichia coli*: the role of antibiotics and the bacterial SOS response. Emerging Infectious Diseases 6, 458–465. 10.3201/eid0605.000503 PMC2627954 10998375

[18] Liu YY (2016) Emergence of plasmid-mediated colistin resistance mechanism MCR-1 in animals and human beings in China: a microbiological and molecular biological study. The Lancet Infectious Diseases 16, 161–168. 26603172 10.1016/S1473-3099(15)00424-7

[19] Hernández M (2017) Co-occurrence of colistin-resistance genes *mcr*-1 and *mcr*-3 among multidrug-resistant *Escherichia coli* isolated from cattle, Spain, September 2015. Eurosurveillance 22, 30586. 28797328 10.2807/1560-7917.ES.2017.22.31.30586 PMC5553059

[20] Threlfall EJ (2000) The emergence and spread of antibiotic resistance in food-borne bacteria. International Journal of Food Microbiology 62, 1–5. 11139009 10.1016/s0168-1605(00)00351-2

[21] Hurtado A, Ocejo M and Oporto B (2017) *Salmonella* spp. and *Listeria monocytogenes* shedding in domestic ruminants and characterization of potentially pathogenic strains. Veterinary Microbiology 210, 71–76. 29103699 10.1016/j.vetmic.2017.09.003

[22] Nielsen EM and Andersen MT (2003) Detection and characterization of verocytotoxin-producing *Escherichia coli* by automated 5′ nuclease PCR assay. Journal of Clinical Microbiology 41, 2884–2893. 12843017 10.1128/JCM.41.7.2884-2893.2003 PMC165313

[23] Perelle S (2004) Detection by 5′-nuclease PCR of Shiga-toxin producing *Escherichia coli* O26, O55, O91, O103, O111, O113, O145 and O157:H7, associated with the world's most frequent clinical cases. Molecular and Cellular Probes 18, 185–192. 15135453 10.1016/j.mcp.2003.12.004

[24] Wang G, Clark CG and Rodgers FG (2002) Detection in *Escherichia coli* of the genes encoding the major virulence factors, the genes defining the O157:H7 serotype, and components of the type 2 Shiga toxin family by multiplex PCR. Journal of Clinical Microbiology 40, 3613–3619. 12354854 10.1128/JCM.40.10.3613-3619.2002 PMC130888

[25] Sánchez S (2015) Development of three multiplex PCR assays targeting the 21 most clinically relevant serogroups associated with Shiga toxin-producing *E. coli* infection in humans. PLoS ONE 10, e0117660. 25629697 10.1371/journal.pone.0117660 PMC4309606

[26] Paton AW and Paton JC (1998) Detection and characterization of Shiga toxigenic *Escherichia coli* by using multiplex PCR assays for *stx*1, *stx*2, *eae*A, enterohemorrhagic *E. coli hly*a, *rfb*O111, and *rfb*O157. Journal of Clinical Microbiology 36, 598–602. 9466788 10.1128/jcm.36.2.598-602.1998 PMC104589

[27] CLSI (2018) Performance Standards for Antimicrobial Susceptibility Testing, 28th edn Wayne, PA, ed: Clinical and Laboratory Standards Institute.

[28] Cochran WG (1954) Some methods for strengthening the common *χ* 2 tests. Biometrics 10, 417.

[29] Venegas-Vargas C (2016) Factors associated with Shiga toxin-producing *Escherichia coli* shedding by dairy and beef cattle. Applied and Environmental Microbiology 82, 5049–5056. 27342555 10.1128/AEM.00829-16 PMC4968536

[30] Cobbaut K (2009) *Escherichia coli* O157 prevalence in different cattle farm types and identification of potential risk factors. Journal of Food Protection 72, 1848–1853. 19777885 10.4315/0362-028x-72.9.1848

[31] Cobbold RN (2004) Comparison of shiga-toxigenic *Escherichia coli* prevalences among dairy, feedlot, and cow-calf herds in Washington state. Applied and Environmental Microbiology 70, 4375–4378. 15240323 10.1128/AEM.70.7.4375-4378.2004 PMC444803

[32] Callaway T (2013) Shiga toxin-producing *Escherichia coli* (STEC) ecology in cattle and management based options for reducing fecal shedding. Agriculture, Food and Analytical Bacteriology 3, 39–69.

[33] Huston CL (2002) Prevalence of fecal shedding of *Salmonella* spp. in dairy herds. Journal of the American Veterinary Medical Association 220, 645–649. 12418525 10.2460/javma.2002.220.645

[34] Davison H (2006) Risk factors associated with the salmonella status of dairy farms in England and Wales. Veterinary Record 159, 871–880. 17189598

[35] Griffin P and Tauxe R (1991) The epidemiology of infections caused by *Escherichia coli* O157:H7, other enterohemorrhagic *E. coli*, and the associated hemolytic uremic syndrome. Epidemiologic Reviews 13, 60–98. 1765120 10.1093/oxfordjournals.epirev.a036079

[36] Monagan Á (2011) Serotypes and virulence profiles of non-O157 shiga toxin-producing *Escherichia coli* isolates from bovine farms. Applied and Environmental Microbiology 77, 8662–8668. 22003024 10.1128/AEM.06190-11 PMC3233096

[37] van Duynhoven YTHP (2008) Prevalence, characterisation and clinical profiles of Shiga toxin-producing *Escherichia coli* in The Netherlands. Clinical Microbiology and Infection 14, 437–445. 18325039 10.1111/j.1469-0691.2008.01963.x

[38] Vally H (2012) Epidemiology of Shiga toxin producing *Escherichia coli* in Australia, 2000–2010. BMC Public Health 12, 63. 22264221 10.1186/1471-2458-12-63 PMC3398300

[39] EFSA (European Food Safety Authority) and ECDC (European Centre for Disease P and C (2017) The European Union summary report on trends and sources of zoonoses, zoonotic agents and food-borne outbreaks in 2016. The EFSA Journal 15, 5077. 10.2903/j.efsa.2017.5077 PMC7009962 32625371

[40] Cabal A (2016) Molecular characterization and antimicrobial resistance of STEC strains isolated from healthy cattle in 2011 and 2013 in Spain. Epidemiology and Infection 144, 2956–2966. 27387818 10.1017/S0950268816001370 PMC9150403

[41] Boerlin P (1999) Associations between virulence factors of Shiga toxin-producing *Escherichia coli* and disease in humans. Journal of Clinical Microbiology 37, 497–503. 9986802 10.1128/jcm.37.3.497-503.1999 PMC84443

[42] Urdahl AM (2003) Animal host associated differences in Shiga toxin-producing *Escherichia coli* isolated from sheep and cattle on the same farm. Journal of Applied Microbiology 95, 92–101. 12807458 10.1046/j.1365-2672.2003.01964.x

[43] Ennis C, McDowell D and Bolton DJ (2012) The prevalence, distribution and characterization of Shiga toxin-producing *Escherichia coli* (STEC) serotypes and virulotypes from a cluster of bovine farms. Journal of Applied Microbiology 113, 1238–1248. 22862826 10.1111/j.1365-2672.2012.05421.x

[44] Krause M, Barth H and Schmidt H (2018) Toxins of locus of enterocyte effacement-negative Shiga toxin-producing *Escherichia coli*. Toxins 10, 241. 10.3390/toxins10060241 PMC6024878 29903982

[45] EFSA (2013) Scientific opinion on VTEC-seropathotype and scientific criteria regarding pathogenicity assessment. The EFSA Journal 11, 3138.

[46] Mora A (2005) Antimicrobial resistance of Shiga toxin (verotoxin)-producing *Escherichia coli* O157:H7 and non-O157 strains isolated from humans, cattle, sheep and food in Spain. Research in Microbiology 156, 793–806. 15921895 10.1016/j.resmic.2005.03.006

[47] European Medicines Agency (2017) Sales of veterinary antimicrobial agents in 30 European countries in 2015. European Surveillance of Veterinary Antimicrobial Consumption (EMA/184855/2017).

[48] Gupta K (2011) International clinical practice guidelines for the treatment of acute uncomplicated cystitis and pyelonephritis in women: a 2010 update by the infectious diseases society of America and the European society for microbiology and infectious diseases. Clinical Infectious Diseases 52, e103–e120. 21292654 10.1093/cid/ciq257

