# Zoonotic approach to Shiga toxin-producing *Escherichia coli*: integrated analysis of virulence and antimicrobial resistance in ruminants and humans – ERRATUM

**DOI:** 10.1017/S0950268820000357

**Published:** 2020-02-20

**Authors:** B. Oporto, M. Ocejo, M. Alkorta, J. M. Marimón, M. Montes, A. Hurtado

During the proofing stage for the above article, Table 3 was modified incorrectly. It should have appeared as below:

**Table 3.** Resistance (%) and distribution of MICs for the 106 ruminant STEC and 36 human STEC (O111/O157) isolates.

Antimicrobial class	Antimicrobial agent	Source	% Resistance^a^	No. of isolates at the indicated MIC (mg/l)																
				0.015	0.03	0.06	0.12	0.25	0.5	1	2	4	8	16	32	64	128	256	512	1024
Aminoglycoside	**Gentamicin**	Ruminants	0.0						83	20	3									
		Humans	2.8							35					**1**					
*β*-Lactam	**Ampicillin**	Ruminants	2.8								17	85	1				**3**			
		Humans	13.9								16	14	1			**5**				
Cephalosporin 3rd	**Cefotaxime**	Ruminants	0.0					106												
		Humans	0.0							36										
Cephalosporin 3rd	**Ceftazidime**	Ruminants	0.0						106											
		Humans	0.0							36										
Quinolone	**Nalidixic acid**	Ruminants	0.0									106								
		Humans	5.6								33	1				**2**				
(Fluoro)quinolone	**Ciprofloxacin**	Ruminants	0.0	35	71															
		Humans	0.0					36												
Sulfonamide	**Sulfamethoxazole**^**b**^	Ruminants	2.8										33	64	6					**3**
Folate pathway inhibitor	**Trimethoprim**	Ruminants	2.8					72	21	9	1					**3**				
Folate pathway inhibitor / Sulfonamide	**Trimethoprim/ Sulfamethoxazole**^c^	Humans	16.7							30				6						
Carbapenem	**Meropenem**	Ruminants	0.0		105	1														
	**Imipenem**	Humans	0.0					35	1											
	**Ertapenem**	Humans	0.0						36											
Glycylcycline	**Tigecycline**	Ruminants	0.0					99	7											
Macrolide	**Azithromycin**^b^	Ruminants	0.0								1	54	50	1						
Miscellaneous	**Chloramphenicol**	Ruminants	0.0										102	4						
Polymyxin	**Colistin**	Ruminants	0.0							105	1									
Tetracycline	**Tetracycline**	Ruminants	2.8								48	53	2				**3**			
*β*-Lactam + *β*-lactamase inhibitor	**Amoxicillin/clavulanic**	Humans	2.8^**d**^								30	3	2	1						
Cephalosporin 2nd	**Cefuroxime**	Humans	0.0									36								
Cephalosporin 2nd	**Cefoxitin**	Humans	0.0									32	4							
Cephalosporin 4th	**Cefepime**	Humans	0.0							36										
Aminoglycoside	**Tobramycin**	Humans	2.8^**d**^							35			1							
Organophosphonate	**Fosfomycin**	Humans	0.0											36						
Nitrofuran	**Nitrofurantoin**	Humans	0.0											32	4					

White fields denote range of dilutions tested for each antimicrobial agent. MICs equal to or above the range are given as the concentration closest to the range and indicated in bold. MICs equal to or lower than the lowest concentration tested are given as the lowest tested concentration. Vertical lines indicate cut-off values: EUCAST epidemiological cut-offs (for ruminant isolates) are represented by thicker lines; CLSI clinical cut-offs (for human isolates) in thin lines, dashed for intermediate and continued for resistant.

a All resistant isolates belonged to serotype O157:H7.

b No cut-off value given by EUCAST; reference as indicated by double vertical lines were used.

c Trimethoprim/Sulfamethoxazole cut-off values are expressed as Trimethoprim concentration (range 1:19–16:304).

d Intermediate resistance.
